# Risk and Ambiguity in Information Seeking: Eye Gaze Patterns Reveal Contextual Behavior in Dealing with Uncertainty

**DOI:** 10.3389/fpsyg.2016.01790

**Published:** 2016-11-17

**Authors:** Peter Wittek, Ying-Hsang Liu, Sándor Darányi, Tom Gedeon, Ik Soo Lim

**Affiliations:** ^1^Instituto de Ciencias Fotónicas-The Institute of Photonic Sciences, Barcelona Institute of Science and TechnologyBarcelona, Spain; ^2^Swedish School of Library and Information Science, University of BoråsBorås, Sweden; ^3^School of Information Studies, Charles Sturt UniversityWagga Wagga, NSW, Australia; ^4^Research School of Computer Science, Human-Centered Computing, The Australian National UniversityActon, ACT, Australia; ^5^School of Computer Science, Bangor UniversityBangor, UK

**Keywords:** information foraging, quantum decision theory, risk and ambiguity minimization, eye gaze, information seeking, cognitive style

## Abstract

Information foraging connects optimal foraging theory in ecology with how humans search for information. The theory suggests that, following an information scent, the information seeker must optimize the tradeoff between exploration by repeated steps in the search space vs. exploitation, using the resources encountered. We conjecture that this tradeoff characterizes how a user deals with uncertainty and its two aspects, risk and ambiguity in economic theory. Risk is related to the perceived quality of the actually visited patch of information, and can be reduced by exploiting and understanding the patch to a better extent. Ambiguity, on the other hand, is the opportunity cost of having higher quality patches elsewhere in the search space. The aforementioned tradeoff depends on many attributes, including traits of the user: at the two extreme ends of the spectrum, analytic and wholistic searchers employ entirely different strategies. The former type focuses on exploitation first, interspersed with bouts of exploration, whereas the latter type prefers to explore the search space first and consume later. Our findings from an eye-tracking study of experts' interactions with novel search interfaces in the biomedical domain suggest that user traits of cognitive styles and perceived search task difficulty are significantly correlated with eye gaze and search behavior. We also demonstrate that perceived risk shifts the balance between exploration and exploitation in either type of users, tilting it against vs. in favor of ambiguity minimization. Since the pattern of behavior in information foraging is quintessentially sequential, risk and ambiguity minimization cannot happen simultaneously, leading to a fundamental limit on how good such a tradeoff can be. This in turn connects information seeking with the emergent field of quantum decision theory.

## 1. Introduction

Foraging is a common pattern of behavior: humans and animals share dedicated cognitive mechanisms to find resources in the environment. Amenities such as food are distributed in spatially localized patches where the task is to maximize one's intake, that is, knowing when to exploit a local patch vs. when it is time to move on and explore one's broader surroundings.

In humans, the underlying neuropsychological mechanisms result in cognitive searches, such as recalling words from memory (Hills et al., 2012, 2015). As part of users' information seeking behavior, the concept of information foraging describes the above quest by a similar strategy (Pirolli and Card, 1999).

Key to the understanding of decisions by a consumer of information is that they are subject to uncertainty: his or her knowledge of the environment is incomplete, so the resulting decisions must go back to perceptions and certain heuristics. By turning to classical works in economy, we can identify two facets of this uncertainty, namely risk and ambiguity (Knight, 1921; Ellsberg, 1961). Their interpretation according to the foraging scenario is in place here.

Briefly, *risk* is the quality of the current patch and our fragmented perception of it. Is the place of good quality? Should one stay here or move on? Since we are already at the preselected location, we do have prior information about it. A risk-minimizing behavior will favor exploitation over exploration, staying longer at individual locations, potentially losing out if outstanding patches remain unvisited.

The above immediately have anthropological overtones. Foraging behavior seems to apply to a much larger domain than just looking for food, such as the optimization of upper and lower extremities of pleasure and pain, gain and loss, benefit and cost, reward and punishment, joy and sorrow. Seeking one while avoiding the other is the subject of risk analysis, where the nature of risk is hesitation. It is obvious that if we are too quick or too slow, we lose a positive option by gaining a negative score somewhere else without even having noticed.

*Ambiguity*, on the other hand, is related to opportunity cost, the price of not foraging elsewhere. “Elsewhere” refers to the rest of the unknown distribution which is not observed at the moment. A human forager who wants to reduce ambiguity first will jump around different patches and explore more, learning as much as possible about the information distribution while reducing the associated uncertainty. This behavior will not stop at the first good enough patch.

To continue the anthropological implications, ambiguity would also mean that all of the above are the essence of situations, of problem solving in general, but any decision we make (and the crucial belief that thereby we have resolved the problem) results in a new situation by trying to escape one. So in a sense, risk would belong to the surface layer and ambiguity to the deep layer of any decision situation. If the above hold, we could identify many more scenarios relevant from psychology to decision theory and from cognitive science to the stock exchange.

Resonating with the aforementioned, our working hypothesis below will be that if animal foraging is subject to uncertainty, and information seeking is an essentially identical activity in a different context, then a limit to simultaneous risk and ambiguity minimization must apply to information foraging as well. This limit emerges from the sequential and incompatible nature of the decisions made to minimize these two aspects of uncertainty. The incompatible decisions are similar to measurements in quantum mechanics where they give rise to the uncertainty principle; thus our work connects information foraging and information seeking behavior to the thriving field of quantum decision theory (Yukalov and Sornette, 2008; Bruza et al., 2009; Khrennikov, 2010; Busemeyer and Bruza, 2012; Ashtiani and Azgomi, 2015). We will demonstrate our point on eye tracking studies data in our analysis of user interactions with novel search interfaces for biomedical information search.

After an overview of the origins and application areas of uncertainty, we provide background information regarding the concept and its relationship with foraging decisions, and the connections between information foraging and information seeking. A user experiment with eye trackers in the context of information search is offered to demonstrate uncertainty as a composite of risk and ambiguity. The eye gaze patterns exhibited by users with different cognitive styles and their search behavior are discussed, and future research is provided.

## 2. Background

### 2.1. The origins and application areas of uncertainty

A decision in the presence of uncertainty means that the outcome cannot be fully predicted before the decision is made. Multiple possible outcomes can occur, and our knowledge of the probability distribution only allows for a limited characterization of uncertainty. Following Knight (1921), Ellsberg (1961), and Camerer and Weber (1992), we can distinguish between two fundamental aspects of uncertainty, aforementioned ambiguity and risk. The simple definition of risk is uncertainty with known probabilities, a certain a priori probability for a given outcome. Ambiguity is also probabilistic but less well defined, generally associated with events that the decision maker has even less information about than the risk of outcomes. The two aspects are also called expected and unexpected uncertainty. Dealing with unexpected uncertainty involves a more subjective evaluation of probabilities. In the case of ambiguity, less information is available, and expected utility is harder to estimate. Not knowing crucial information, such as the probability distribution of the outcomes, is a frightening prospect which explains why most people are ambiguity-averse (Ellsberg, 1961). The two forms of uncertainty are so different that dealing with risk and ambiguity are supported by distinct neural mechanisms in humans (Huettel et al., 2006).

Apart from this probabilistic nature of decisions in an uncertain environment, there is an even deeper form of uncertainty: the kind we normally refer to in the context of quantum mechanics. Some measurements on a quantum system are simply incompatible: measuring one aspect of the system prevents us from learning more about another aspect thereof, explored by a different measurement—this is known as the Heisenberg uncertainty principle (Heisenberg, 1927). Using the formalism of quantum mechanics in decision theory is not novel either: recently there has been a surge of interest in this topic (Bruza et al., 2009; Khrennikov, 2010; Busemeyer and Bruza, 2012; Wittek et al., 2013).

### 2.2. Uncertainty and foraging decisions

We are especially interested in how risk and ambiguity appear in sequential decisions. Simultaneous or coordinated decision making, on the other hand, is more complex, being less common among animals because it involves comparative evaluation. Pointing at a major difference between the animal kingdom vs. man, Kolling et al. (2012) showed that humans are able to choose between these two models in uncertain environments. A foraging scenario is a good example of sequential decision making: food resources are available in patches, and a forager must find an optimal strategy to consume the resources. There is a cost associated with switching from one patch to another. Uncertainty relates to the quality of the current patch, the quality of background options—the opportunity cost of not foraging elsewhere–, while the environment is also subject to changes. The forager has to minimize the tradeoff between exploitation of a patch vs. exploration of background options. This pattern is not restricted to food consumption: for instance, it pertains to mate selection, retrieving memories, and consumer decisions. In fact, the same neural mechanism can serve these different functions (Adams et al., 2012).

Optimal foraging theory establishes the strategy to follow if the probabilities can be estimated and updated by the forager (MacArthur and Pianka, 1966; Charnov, 1976). Ambiguity alters the behavior: for example, unexpected forms of uncertainty may trigger more exploration (Cohen et al., 2007). We would like to see how ambiguity and risk can be minimized in sequential decisions, and how that affects exploration and exploitation.

Many decisions require an exploration of alternatives before committing to one, and exploiting the consequences thereof. This is known as foraging in animals that face an environment in which food resources are available in patches: the forager explores the environment looking for high-quality patches, eventually exploiting a few of them only. Such decisions take place in an uncertain environment: ambiguity about the quality of patches and the risk of not foraging at better patches force the forager to accept a tradeoff.

Risk-sensitive foraging is not exclusive to animals, human subjects also show similar behavioral patterns (Pietras et al., 2003; Rushworth et al., 2012). An optimal solution between exploration and exploitation is generally not known, except in cases with strong assumptions about both the environment and the decision maker (Cohen et al., 2007). The tradeoff between exploration and exploitation is also known as the partial-feedback paradigm, linking the decision model to the description–experience gap (Hertwig and Erev, 2009): people perceive the risk of a rare event differently if the probability distribution is known (decision from description) vs. when they have to rely on more uncertain information (decision from experience).

### 2.3. Information seeking as foraging

To take the next step in our working hypothesis, below we shall look at a scenario where seeking was exercised by gaze fixation at segments of user interfaces with significant elements of content, and show that underlying the seemingly random walks of eye gaze on the screen, there is order in the patterned data inasmuch as a certain typology of user behavior applies to them.

The information foraging nature of the data was recognized by eye tracking analysis, based on the concept of information scent, operationalized as “the proportion of participants who correctly identified the location of the task answer from looking at upper branches in the tree” in a study of user interactions with visualization of large tree structures (Pirolli et al., 2000). Pirolli et al. (2001) provided further theoretical accounts for scanpaths from cognitive perspectives in which users were able to find information more quickly when strong information scent was detected. Chi et al. (2001) built a computational model for user information needs and search behavior based on information scent, and the model and algorithm were evaluated by simulated studies. More recently, the modeling of user search behavior using eye tracking techniques has focused on levels of domain knowledge, user interests, types of search task and relevance judgments in search processes (Cole et al., 2010, 2013; Gwizdka, 2014; Vakkari et al., 2014; Zhang et al., 2015). However, there is still limited understanding of the effect of individual differences and user perceptions of search tasks on eye gaze patterns in information search. White (2016a,b) provided a review of information foraging and user interactions with search systems.

The eye gaze patterns, an indicator of user attention and cognitive processes have been extensively studied for designing user interfaces, such as the functional grouping of interface menu (Goldberg and Kotval, 1999; Brumby and Zhuang, 2015), faceted search interface (Kules et al., 2009; Kemman et al., 2013) and comparison of interface layouts (Kammerer and Gerjets, 2012). Information retrieval researchers have been concerned with users' attention to the ranking position of documents and different components of search engine results page (SERP) (Cutrell and Guan, 2007; Lorigo et al., 2008; Dumais et al., 2010; Savenkov et al., 2011; Kim et al., 2016). These studies generally suggest that there is no significant difference in users' eye gaze patterns on comparisons of search interface layouts, and users' attention to elements of interfaces depends on the length and quality of snippets on SERPs, as well as the displayed position of search results.

## 3. User experiment

A user experiment was designed to investigate user eye gaze and search behavior in biomedical search tasks, with particular reference to different elements of search interfaces (i.e., Medical Subject Headings (MeSH) terms, title, authors, and abstract).

We used a 4×4×2 factorial design with four search interfaces, controlled search topic pairs and cognitive styles. A 4×4 Graeco-Latin square design was used (Fisher, 1935) to arrange the experimental conditions. Each user was assigned 8 topics in total, with a 7-min limit for each topic, and the experiment took about 90 min in total. Refer to Video S1 in the Supplementary Material for a demonstration.

This study was carried out in accordance with the recommendations of National Statement on Ethical Conduct in Human Research, The National Health and Medical Research Council, with written informed consent from all subjects. All subjects gave written informed consent. The protocol was approved by the The Science & Medical Delegated Ethics Review Committee, The Australian National University, and the Human Research Ethics Committee, Charles Sturt University.

A information sheet regarding the nature and procedures of the research project was explained to the participant. A consent form was signed with the conditions of (1) freely given consent; (2) engagement with searching an experimental system and collection of background information, search experiences and individual differences, such as cognitive styles; (3) confidentiality of personal information; (4) the consent form and any other identifying materials will be stored separately; (5) withdrawal from the research project at any time; and (6) participation is completely voluntary.

### 3.1. Research hypotheses

This study has been informed by uncertainty and foraging decisions in information seeking, since patches of information can be found in different elements of search interfaces in potentially uncertain search environments. The hypothesis that unexpected forms of uncertainty may trigger more exploratory behavior can be approached from user perceptions of task difficulty in information search (Cohen et al., 2007). Exploitation of a patch vs. exploration of background options in foraging can be observed by eye gaze and user search behaviors, and explained by the concept of information scent (Chi et al., 2001; Pirolli et al., 2001).

Based on the concepts of uncertainty and foraging decisions and their relationship to information seeking, we propose four research hypotheses in this study:

H1: Users will attend to different elements of search interfaces when they are under perceived difficult search environments.
H2: User traits of cognitive styles will affect information seeking strategies in terms of eye gaze behavior.
H3: Search behavior associated with expanding mental efforts will change in uncertain environments.
H4: Search behavior types that involve notable mental efforts and exploitation of resources are correlated with changes in eye gaze patterns.

### 3.2. Search interfaces

Participants searched on four different search interfaces, with a single search system behind the scenes. The four search interfaces were distinguished by whether MeSH terms were presented and how the displayed MeSH terms were generated, partly because we were concerned with how users search for patches of information, using different information seeking strategies such as exploitation of a patch vs. exploration of background options. The complexity of interface design characterized different perceptions of risk of information patches.

**Figure 1:**
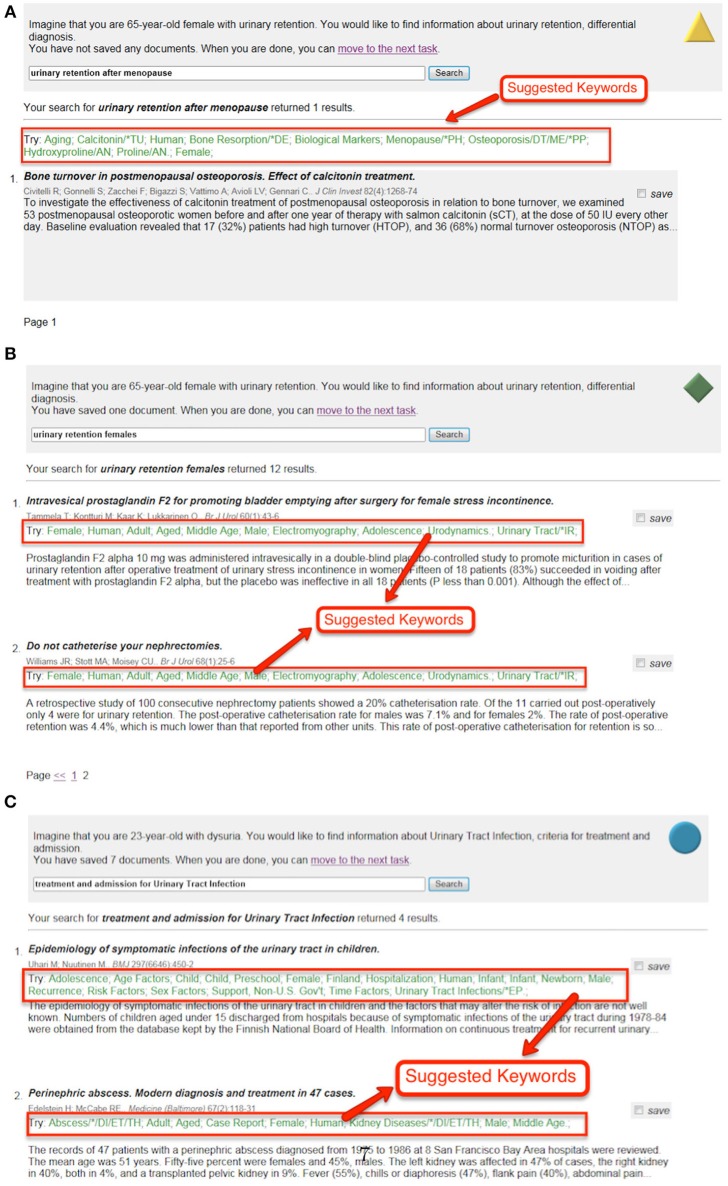
**Three of the four search interfaces in the study**. **(A)** Screenshot of Interface “B,” suggestions per-query and displayed at top. **(B)** Screenshot of Interface “C,” suggestions per-query and displayed at top. **(C)** Screenshot of Interface “D,” suggestions per-document and displayed with the document.

**Interface “A”** mimicked web search and other search systems with no controlled vocabulary. This interface had a brief task description at top; a conventional search box and button; and each result was represented with its title, authors, publication details, and abstract where available.
  Full text was not available, so the results were not clickable. Users judged their success on the titles and abstracts alone.
**Interface “B”** (Figure 1A) added MeSH terms to the interface. After the user's query was run, MeSH terms from all results were collated; the ten most frequent were displayed at the top of the screen. This mimics the per-query suggestions produced by systems like ProQuest^1^.
  MeSH terms were introduced with “Try:” and were clickable: if a user clicked a term, his or her query was refined to include the MeSH term and then re-run. It was hoped that the label, and the fact they work as links, would encourage users to interact with them.
**Interface “C”** (Figure 1B) used the same MeSH terms as “B” but displayed them alongside each document, where they may have been more (or less) visible. It is a hybrid of interfaces “B” and “D.”
**Interface “D”** mimicked EBSCOhost^2^ and similar systems that provide indexing terms alongside each document. As well as the standard elements from interface “A,” interface “D” displayed the MeSH terms associated with each document, as part of that document's surrogate (Figure 1C).
  Again, terms were introduced with “Try:” and were clickable.

Each interface was labeled with a simple figure: a square, circle, diamond, or triangle, which was referred to in the exit questionnaire. A save icon alongside each retrieved document was provided to collect user perceived relevant documents.

### 3.3. Search topics

**Figure 2:**
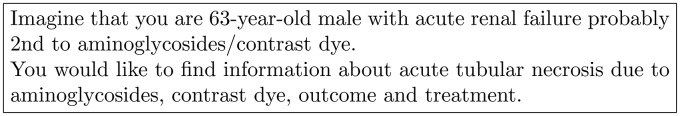
**An example OHSUMED search topic, reworded for the participants**.

The search topics used were a subset of the clinical topics from OHSUMED (Hersh et al., 1994), originally created for information retrieval system evaluation. They were slightly rewritten to read as instructions to the participants (see Figure 2 for an example). Topics were selected to cover a range of difficulties.

### 3.4. Procedure

Participants were given brief instructions about the search task and system features, followed by a practice topic and then the searches proper. They were informed that the test collection is incomplete and out-of-date since the OHSUMED test collection (Hersh et al., 1994) was used, with MEDLINE data from 1987 to 1991. Recorded user interaction data included all queries, mouse clicks, retrieved and saved documents, time spent, and eye movements. Electroencephalogram (EEG) readings were also captured.

Background and exit questionnaires collected demographic information and asked participants about their perception of the search process. Participants' opinions of the tasks and the interfaces were sought. Finally, information on their cognitive styles was also collected by a computerized test (Peterson et al., 2003; Peterson, 2005), which took a further 15 min to complete.

### 3.5. Hardware and software

The search system was built on Solr^3^, with the search results ranked by default relevance score. The MeSH terms were not specifically weighted.

Eye gaze data was recorded from two Sony VFCB-EX480B infrared (IR) cameras which were controlled by Seeing Machines FaceLab 4.5 software^4^ and attached to a dedicated machine running Windows 7. At the same time, EyeWorks Design and EyeWorks Record^5^ were used to present instructions for the corresponding search tasks during the experiment. Gaze points were recorded at 60 Hz, and the eye gaze data included the *x* and *y* coordinates of where the eye was looking on the screen, as well as the time of that recording. EEG data were recorded with an Emotiv headset^6^ to monitor brain activities throughout the search session. A Windows 7 computer was dedicated to the cognitive styles test.

### 3.6. Data analysis

Recordings were analyzed to see how often there were fixations in different parts of document surrogates (i.e., different elements of the interfaces), and therefore how often people looked at each part.

Four common areas of interest (AOI) were specified: title, author, abstract and MeSH (except for Interface A, without MeSH) to investigate which elements received attention. EyeWorks Analyze^7^ was used to specify the AOI, and fixations were specified as gazes within a 5-pixel radius which lasted at least 75 ms (Marshall, 2000). The EEG data was not included since our analysis was focused on eye gaze and search behaviors.

Since we were interested in the relationship between user perceptions of search tasks vs. system features, and eye gaze behavior, the data were analyzed by a logarithmic cross-ratio analysis (Fleiss et al., 2003) for dichotomous outcomes. The independent variables of user traits of cognitive styles and perceived search task difficulty, as well as dependent variables of search behavior and eye gaze measures, were converted into binary variables for further analysis. This data analysis technique was chosen because it is resistant to sample selection bias and applied in the study of the relationship between user traits and search performance (Saracevic et al., 1988).

In the study a post-search questionnaire was used to assess user perceptions about the search processes, in which search task difficulty was also identified as important moderator of eye gaze behavior (Toker et al., 2013). No follow-up interview was conducted in this study. As such, our data analysis was focused on information search in a difficult environment with a high degree of perceived uncertainty.

## 4. Results of search behavior and eye gaze

Overall, our results support H1, H2, and H4, indicating significant significant effects on eye gaze behavior in terms of proportion of fixations in reading time. This in turn translates to different strategies in dealing with risk and ambiguity.

### 4.1. Search task difficulty and eye gaze

Table 1 reveals that there was a statistically significant relationship between user perception of search task difficulty and proportion of fixations in reading time on all elements in interfaces. One-way ANOVA indicates a significant interaction effect of interface and task difficulty on the fixations time spent in title [*F*_(3, 248)_ = 3.72, *p* < 0.05] and MeSH terms [*F*_(3, 248)_ = 3.71, *p* < 0.05], but it is not the case for the element of author [*F*_(3, 248)_ = 1.69, *p* > 0.05] and abstract [*F*_(3, 248)_ = 1.55, *p* > 0.05]. These results support our first research hypothesis that users will attend to different elements of search interfaces, when they are under perceived difficult search environments.

**Table 1:** **Summary of the relationship between search task difficulty and eye gaze (N search task difficulty = 256, N eye gaze = 256; statistical significance at 95%)**.

	**Cut point (Mean)**	**Odds ratio**	**Log odds**	**Stand error**.	***t*-value**	**Stat. signif**.
**Areas of interest**						
Title	24.33	0.06	−2.73	0.71	−3.86	Yes
Author	12.53	0.12	−2.13	0.70	−3.02	Yes
Abstract	45.81	0.13	−2.05	0.71	−2.90	Yes
MeSH	17.34	0.07	−2.72	0.70	−3.87	Yes

### 4.2. Search task difficulty, cognitive style and eye gaze

In the study the E-CSA-WA (Extended Cognitive Style Analysis–Wholistic Analytic) test was used to determine user's cognitive style. A Wholistic Analytic ratio (WA ratio) for each participant was produced (Peterson et al., 2003). The results suggest that there was no significant relationship between the users' cognitive style and eye gaze across all elements in interfaces in terms of proportion of fixations in reading time.

One-way ANOVA of the effects of search task difficulty, search interface and cognitive style and their interactions on eye gaze indicates significant interaction effects of difficulty and cognitive style [*F*_(1, 240)_ = 4.54, *p* < 0.05], and cognitive style vs. search interface [*F*_(3, 240)_ = 2.89, *p* < 0.05] in terms of fixation time on the element of abstract. We found significant interaction effects between search task difficulty and search interface [*F*_(3, 240)_ = 4.19, *p* < 0.01], and search interface and cognitive style [*F*_(1, 240)_ = 4.24, *p* < 0.01] for the element of MeSH terms. These results suggest that *searchers with different cognitive styles may use different search strategies under an environment with uncertainty perceived as difficult and observed by their eye gaze behavior*. Our second research hypothesis that user traits of cognitive styles will affect information seeking strategies in eye gaze behavior is supported.

### 4.3. Search task difficulty and search behavior

Table 2 shows that when search tasks were perceived difficult, users tended to spend less time searching, issued less queries or typed queries, saved fewer documents and had fewer mouse clicks, but there was no difference in the number of MeSH queries issued and the number of pages viewed.

**Table 2:** **Summary of the relationship between search task difficulty and search behavior (N search task difficulty = 256, N eye gaze = 256; statistical significance at 95%)**.

	**Cut point (Mean)**	**Odds ratio**	**Log odds**	**Stand error**.	***t*- value**	**Stat. signif**.
**Search behavior**						
Time spent	185.7	0.11	−2.25	0.70	−3.20	Yes
Number of queries issued	3.80	0.12	−2.13	0.72	−2.98	Yes
Number of MeSH queries issued	0.33	0.30	−1.19	0.74	−1.60	No
Number of typed queries issued	3.48	0.18	−1.73	0.74	−2.33	Yes
Number of pages viewed	5.04	0.23	−1.47	0.76	−1.94	No
Number of saved documents	3.63	0.10	−2.33	0.71	−3.26	Yes
Number of mouse clicks	4.88	0.09	−2.42	0.72	−3.37	Yes

Overall, the results indicate that searchers made less mental effort when the search tasks were difficult, and they tended to optimize limited resources in information seeking, demonstrated both by eye gaze (Table 1) and search behavior (Table 2). *Search behavior associated with expending mental efforts like issuing MeSH terms and viewing SERPs has not changed according to the uncertainty within the environment, such as perceived search task difficulty*. Therefore, our third research hypothesis that search behavior associated with expanding mental efforts will change in uncertain environments is not supported.

### 4.4. Search behavior and eye gaze in information search

*Number of queries issued*. Table 3 shows that there was a statistically significant relationship between the number of queries issued and the area of interest (AOI) of MeSH terms. In other words, when users issued more queries, they paid significantly more attention to the MeSH terms in search results. This might indicate that processes of query reformulations are very resource intensive, particularly for using MeSH terms.

**Table 3:** **Summary of the relationship between number of queries issued and gaze (N number of queries issued = 256, N eye gaze = 256; statistical significance at 95%)**.

	**Cut point (Mean)**	**Odds ratio**	**Log odds**	**Stand error**.	***t*- value**	**Stat. signif**.
**Areas of interest**						
Title	24.33	0.76	−0.27	0.25	−1.09	No
Author	12.53	0.93	−0.07	0.25	−0.28	No
Abstract	45.81	0.92	−0.09	0.25	−0.34	No
MeSH	17.34	1.67	0.51	0.25	2.04	Yes

*Number of MeSH queries issued*. Table 4 reveals that there was a statistically significant relationship between the number of MeSH queries issued and the element of abstract viewed. That is, when users issued more MeSH queries, they paid significantly less attention to the abstract in interfaces. This might reflect the fact that using MeSH terms can be more efficient in the search processes.

**Table 4:** **Summary of the relationship between number of MeSH queries issued and gaze (N number of MeSH queries issued = 256, N eye gaze = 256; statistical significance at 95%)**.

	**Cut point (Mean)**	**Odds ratio**	**Log odds**	**Stand error**.	***t*- value**	**Stat. signif**.
**Areas of interest**						
Title	24.33	1.39	0.33	0.37	0.89	No
Author	12.53	1.01	0.01	0.37	0.03	No
Abstract	45.81	0.45	−0.80	0.39	−2.02	Yes
MeSH	17.34	1.77	0.57	0.38	1.50	No

*Number of mouse clicks*. Table 5 indicates a statistically significant relationship between the number of mouse clicks and the element of title visited. That is, users who clicked the mouse more often were less likely to be interested in the titles.

**Table 5:** **Summary of the relationship between number of mouse clicks and gaze (N number of mouse clicks = 256, N eye gaze = 256; statistical significance at 95%)**.

	**Cut point (Mean)**	**Odds ratio**	**Log odds**	**Stand error**.	***t*- value**	**Stat. signif**.
**Areas of interest**						
Title	24.33	0.46	−0.77	0.26	−3.00	Yes
Author	12.53	0.95	−0.05	0.25	−0.21	No
Abstract	45.81	1.21	0.19	0.25	0.76	No
MeSH	17.34	1.19	0.17	0.25	0.68	No

*Number of pages viewed*. Table 6 brings evidence for the same inverse relationship between the number of pages viewed vs. titles inspected.

**Table 6:** **Summary of the relationship between number of pages viewed and gaze (N number of pages viewed = 256, N eye gaze = 256; statistical significance at 95%)**.

	**Cut point (Mean)**	**Odds ratio**	**Log odds**	**Stand error**.	***t*- value**	**Stat. signif**.
**Areas of interest**						
Title	24.33	0.47	−0.75	0.27	−2.82	Yes
Author	12.53	0.61	−0.49	0.26	−1.85	No
Abstract	45.81	1.40	0.34	0.26	1.31	No
MeSH	17.34	1.54	0.43	0.26	1.65	No

*Number of documents saved*. Table 7 reveals that there was a statistically significant relationship as regards the number of documents saved vs. abstracts and MeSH terms as document segments inspected: when users saved more documents, they devoted significantly more attention to the element of abstract, but less attention to the MeSH terms.

**Table 7:** **Summary of the relationship between number of documents saved and gaze (N number of documents saved = 256, N eye gaze = 256; statistical significance at 95%)**.

	**Cut point (Mean)**	**Odds ratio**	**Log odds**	**Stand error**.	***t*- value**	**Stat. signif**.
**Areas of interest**						
Title	24.33	1.09	0.08	0.25	0.32	No
Author	12.53	1.32	0.28	0.26	1.08	No
Abstract	45.81	1.72	0.54	0.26	2.10	Yes
MeSH	17.34	0.38	−0.97	0.26	−3.70	Yes

*Summary of search behavior and gaze pattern types*. Table 8 provides a summary of search behaviors and gaze patterns. These results clearly show that types of searching behavior, such as issuing queries with MeSH terms that imply notable mental effort and strive at the exploitation of resources, are correlated with changes in eye gaze patterns. Therefore, our fourth research hypothesis that search behavior that involve with notable mental efforts and exploitation of resources are correlated with changes in eye gaze patterns is supported.

**Table 8:** **Summary of the relationship between search behavior and gaze patterns**.

	**No. of queries issued**	**No. of MeSH queries issued**	**No. of mouse clicks**	**No. of pages viewed**	**No. of documents saved**
Title	—	—	❍	❍	—
Author	—	—	—	—	—
Abstract	—	❍	—	—	●
MeSH	●	—	—	—	❍

*The relationship is not statistically significant (—), positively significant (●), or negatively significant (❍)*.

## 5. Results and discussion

We summarize the main findings in the data as follows:

- When users perceived their search tasks as difficult, they did not attend to all content elements in documents. [H1, confirmed]
- Searchers with different cognitive styles may use different search strategies under an environment with uncertainty they perceive as difficult. [H2, confirmed]
- Search behavior associated with expanding mental efforts like issuing MeSH terms and viewing SERPs has not changed according to the uncertainty within the environment, such as perceived search task difficulty. [H3, rejected]
- Certain search behavior types, such as issuing queries and MeSH terms that involve notable mental efforts and exploitation of resources, are correlated with changes in eye gaze patterns. [H4, confirmed]

These findings indicate distinct strategies in dealing with uncertainty, possibly changing from preferring exploration to exploitation and vice versa, and therefore corroborate our hypothesis that the corresponding observations do not commute, which hints at a form of quantum-like behavior.

In the above eye tracking study, the document surrogates and the four layouts characterize different perceptions of risk of information patches, gazing time being a good figure of merit for exploitation. Exploration is the jumping gaze combined with a repeated query as these reduce overall ambiguity. There is evidence that wholistic users prefer to get an overview of tasks before drilling down to detail, whereas analytic users look for specific information. These two extreme user behaviors rely on the two measurement operators, namely risk- vs. ambiguity reduction, in different order, proving non-commutativity. At this point there is no significant relationship between the users' cognitive style and the AOIs though.

However, if we also change the perceived risk by varying the search interface, the picture changes. The effect of cognitive styles, interfaces and their interactions on the AOI of MeSH terms (excluding Interface A) is statistically significant in terms of cognitive style and interface interactions, and weakly significant in terms of cognitive style [*F*_(1, 188)_ = 2.79, *p* < 0.01]. Interfaces make a statistically significant difference for the wholistic style [*F*_(2, 111)_ = 6.58, *p* < 0.001], and cognitive styles make a statistically significant difference in Interface B [*F*_(1, 62)_ = 5.11, *p* < 0.05]. The results indicate that wholistic users' attention to the MeSH terms is more affected by search interfaces than that of analytic users, and this interaction effect is significant when interacting with Interface B. Thus, non-commutative measurements emerge.

To sum up, uncertainty as a composite of risk and ambiguity drives information seeking behavior in a complex way, with successive decisions attempting to minimize both components at the same time. However, to find their joint optimum is not possible, because risk-prone and ambiguity-prone behavior manifest two versions of foraging attitude, called the “consume first and worry later” (exploitation) vs. the “worry first and consume later” (exploration) types. Whichever option taken, it becomes the context of the opposite alternative, so that ambiguity minimization dependent on risk minimization vs. risk minimization dependent on ambiguity minimization yield different sets of retrieved items, i.e., the outcome of information seeking as a process is non-commutative.

For every case where this joint optimum seeking mentality influences the results, plus the decision making process that has led to a particular outcome must be preserved for future reconstruction, our findings are relevant. We have found that the above two types of behavior go back to the application of two operators, risk- and ambiguity-aversion, so that by applying now this, then the other first, their sequential application leads to different results, called non-commutativity. This is related to the uncertainty principle in quantum mechanics, therefore we identify information seeking as another link to quantum decision theory (Wittek et al., 2013; Ashtiani and Azgomi, 2014; Aerts and Sozzo, 2016).

## 6. Conclusions and future research

We interpreted risk and ambiguity as two types of measurement on an uncertain environment, arguing that in an information foraging scenario, these measurements are sequential and do not commute, that is, reversing their order yields different outcomes. We demonstrated this by analyzing user behavior in interacting with different designs of search results, specifically, by tracking the gaze of users. Depending on the degree of uncertainty involved, qualitatively different types of information seeking behavior emerged, agreeing with our hypotheses.

We have reason to believe that similar data, such as clickstreams, will show similar patternedness as evidence of non-commutative user behavior manifesting the same cognitive types in a different setting. In a broader context, noncommuting measurements are standard tools in quantum mechanics, and they are being explored in quantum decision theory for modeling decision problems and known fallacies—our work connects information seeking to this line of research.

## Author contributions

PW and SD participate conceptualization of theoretical framework and paper writing; YL and TG participate conceptualization and execution of eye-tracking user study and paper writing; IL participates paper writing.

### Conflict of interest statement

The authors declare that the research was conducted in the absence of any commercial or financial relationships that could be construed as a potential conflict of interest.

## Footnotes

^1^For example, see http://www.proquest.co.uk/en-UK/products/brands/pl_pq.shtml.

^2^http://www.ebscohost.com/.

^3^http://lucene.apache.org/solr/.

^4^https://www.seeingmachines.com/.

^5^http://www.eyetracking.com/Software/EyeWorks.

^6^http://www.emotiv.com/.

^7^http://www.eyetracking.com/Software/EyeWorks.
